# Occupational Risks and Chronic Obstructive Pulmonary Disease in the Indian Subcontinent: A Critical Review

**DOI:** 10.7759/cureus.41149

**Published:** 2023-06-29

**Authors:** Jijnasha Mishra, Sourya Acharya, Avinash B Taksande, Roshan Prasad, Pratiksha K Munjewar, Mayur B Wanjari

**Affiliations:** 1 Community Medicine, Jawaharlal Nehru Medical College, Datta Meghe Institute of Higher Education and Research, Wardha, IND; 2 Medicine, Jawaharlal Nehru Medical College, Datta Meghe Institute of Higher Education and Research, Wardha, IND; 3 Physiology, Jawaharlal Nehru Medical College, Datta Meghe Institute of Higher Education and Research, Wardha, IND; 4 Internal Medicine, Jawaharlal Nehru Medical College, Datta Meghe Institute of Higher Education and Research, Wardha, IND; 5 Medical-Surgical Nursing, Smt. Radhikabai Meghe Memorial College of Nursing, Datta Meghe Institute of Higher Education and Research, Wardha, IND; 6 Research and Development, Jawaharlal Nehru Medical College, Datta Meghe Institute of Higher Education and Research, Wardha, IND

**Keywords:** collaborative efforts, workforce productivity, occupational health and safety, prevention, prevalence, indian subcontinent, occupational risks, chronic obstructive pulmonary disease (copd)

## Abstract

Chronic obstructive pulmonary disease (COPD) is a significant public health concern in the Indian subcontinent, with high prevalence rates observed in countries like India, Pakistan, Bangladesh, and neighboring nations. This review article critically examines the occupational risks associated with COPD in the region and emphasizes the need for comprehensive preventive strategies. The review begins by providing background on COPD and highlighting its impact on individuals and the economy. It then explores the different occupational hazards that contribute to the development and progression of COPD, including exposure to airborne pollutants and chemicals, occupational dust, and smoking and secondhand smoke in the workplace. The existing occupational health and safety regulations in the Indian subcontinent are assessed, along with an evaluation of their effectiveness in addressing occupational risks for COPD. The review also highlights gaps and challenges in implementing and enforcing these regulations. The impact of COPD on occupational health and the economy is examined, emphasizing the burden it places on affected individuals and their ability to work. The economic implications of COPD-related productivity losses are evaluated, underscoring the importance of addressing occupational risks to improve workforce health and productivity. Prevention and mitigation strategies are explored, including an overview of preventive measures to reduce occupational risks for COPD, the significance of early detection and diagnosis of COPD in the workplace, and the implementation of engineering controls, personal protective equipment, and ventilation systems. The role of education and training programs for workers and employers is also discussed. The review identifies research gaps in the current understanding of occupational risks and COPD in the Indian subcontinent and suggests future research directions to address these gaps. It emphasizes the importance of collaborative efforts between researchers, policymakers, and industry stakeholders to generate evidence, inform policy decisions, and implement effective interventions.

## Introduction and background

Chronic obstructive pulmonary disease (COPD) is a chronic respiratory condition characterized by persistent airflow limitation, which makes breathing difficult. It is a major global health issue, causing significant morbidity, mortality, and economic burden. COPD is primarily associated with risk factors such as smoking, air pollution, and genetic predisposition. However, occupational factors also play a significant role in the development and progression of COPD [1,2].

Occupational risks refer to exposure to hazardous substances and working conditions that can lead to the development or exacerbation of COPD. Several occupational hazards have been identified as contributing factors to COPD. Exposure to airborne pollutants and chemicals, such as dust, gases, fumes, and biological agents, is a common occupational risk factor. Mining, construction, textile manufacturing, and agriculture workers are particularly vulnerable to these exposures. Additionally, exposure to secondhand smoke can further increase the risk of COPD among non-smoking workers [2,3].

The impact of occupational risks on COPD is substantial. Studies have shown that occupational exposures contribute significantly to the burden of COPD worldwide. Occupational COPD often develops after years of exposure to hazardous substances, leading to chronic inflammation and damage to the airways and lung tissue. It affects affected individuals' health and quality of life and has significant economic implications due to decreased work productivity, increased healthcare costs, and disability [3,4].

The Indian subcontinent, comprising countries such as India, Pakistan, Bangladesh, and neighboring regions, has a high burden of COPD. Genetic, environmental, and occupational factors influence the prevalence of COPD in this region. While the impact of smoking and air pollution on COPD has been widely recognized, the specific occupational risks and their contribution to COPD in the Indian subcontinent remain understudied [4,5].

Studying occupational risks for COPD in the Indian subcontinent is paramount for several reasons. Firstly, the region has a large and diverse workforce in various industries prone to occupational hazards. These industries include mining, quarrying, construction, textile and garment manufacturing, and agriculture. Understanding the occupational risks associated with these industries can help develop targeted preventive measures and policies [5,6]. Secondly, the Indian subcontinent faces unique occupational health and safety regulations and enforcement challenges. Assessing the effectiveness of existing regulations and identifying gaps in their implementation can guide policymakers in formulating robust measures to protect workers' respiratory health. Lastly, addressing occupational risks for COPD is crucial for improving occupational health, productivity, and overall public health in the Indian subcontinent. Reducing exposure to hazardous substances in the workplace makes it possible to prevent or mitigate the development and progression of COPD, resulting in a healthier and more productive workforce [2,5,6].

This review article aims to critically examine the literature on occupational risks and COPD in the Indian subcontinent. We will explore the prevalence of COPD, identify specific occupational risks, evaluate the impact of COPD on occupational health and the economy, discuss preventive strategies, and highlight research gaps. By shedding light on this important topic, we hope to contribute to the knowledge base and stimulate further research and actions to protect workers from occupational risks and reduce the burden of COPD in the Indian subcontinent.

## Review

Methodology

This review article employed a comprehensive and systematic approach to gathering relevant information on occupational risks and COPD in the Indian subcontinent. The research methodology involved thoroughly searching the scientific literature and relevant databases to identify studies, research articles, and related reports. We ensured broad and representative coverage of the subject matter by utilizing various sources, including academic journals, conference proceedings, and reputable websites. The authors evaluated a wide range of studies to provide a comprehensive overview. The included studies encompassed different study designs, including observational studies (e.g., cohort studies, case-control studies, cross-sectional studies), experimental studies, and systematic reviews. This allowed us to capture diverse types of evidence and strengthen the reliability of the findings.

Specific selection criteria were applied to ensure the inclusion of high-quality and relevant studies. Firstly, the authors considered studies that directly investigated the association between occupational risks and COPD in the Indian subcontinent, ensuring direct relevance to the research question. Secondly, studies with adequate sample sizes and representative populations were prioritized to enhance the findings' reliability and generalizability. Primary consideration was given to English language studies during the inclusion process. The review focused on studies published within a specific timeframe to ensure the inclusion of recent and up-to-date research. The literature search encompassed studies published from 2000 to the present, allowing the authors to capture the most relevant and current information.

Prevalence of COPD in the Indian subcontinent

COPD is a significant public health issue in the Indian subcontinent, affecting millions of individuals in countries such as India, Pakistan, Bangladesh, and neighboring regions. Various studies have reported high prevalence rates of COPD in this region, highlighting the magnitude of the problem [5,7,8]. In India, for instance, the Burden of Obstructive Lung Disease (BOLD) study estimated the prevalence of COPD to be around 5.3% among adults aged 40 years and above. The Global Burden of Disease Study also highlighted the substantial burden of COPD in India, ranking it as the second leading cause of years lived with disability (YLD) in the country. Similarly, in Pakistan, studies have reported COPD prevalence rates ranging from 4.8% to 6.3% among adults [7,8].

Bangladesh, too, faces a significant burden of COPD. According to the World Health Organization (WHO), COPD is estimated to affect around 6.6% of the adult population in Bangladesh. Additionally, neighboring countries, such as Nepal and Sri Lanka, report considerable COPD prevalence rates [3]. 

Risk Factors Contributing to the High Prevalence of COPD in the Region

Several risk factors contribute to the high prevalence of COPD in the Indian subcontinent. The primary risk factor is tobacco smoking, both active and passive. Smoking rates are high in this region, with many individuals using tobacco products such as cigarettes, bidis, and smokeless tobacco. Prolonged exposure to tobacco smoke damages the lungs and leads to COPD [9,10].

In addition to smoking, indoor and outdoor air pollution is a major risk factor for COPD in the Indian subcontinent. Indoor air pollution arises from burning solid fuels for cooking and heating, which releases harmful pollutants into the air. This exposure is particularly prevalent in rural areas where biomass fuels are commonly used. Outdoor air pollution, driven by industrial emissions, vehicular pollution, and urbanization, also contributes to the burden of COPD in urban areas [11].

Occupational exposures to various hazardous substances and pollutants are another significant risk factor for COPD. Workers in industries such as mining, construction, textile manufacturing, and agriculture are exposed to airborne pollutants, dust, fumes, and chemicals that can damage the lungs over time, leading to the development of COPD [6,9].

Other risk factors for COPD in the region include poor socioeconomic conditions, inadequate access to healthcare, lack of awareness about the disease, and genetic predisposition. Understanding these risk factors and their contributions to the high prevalence of COPD in the Indian subcontinent is crucial for implementing targeted prevention and intervention strategies. Addressing these risk factors can reduce the burden of COPD and improve respiratory health in the region [9-11].

Occupational Risks Associated With COPD

Occupational hazards play a significant role in the development and progression of COPD. Several types of hazards are associated with an increased risk of COPD in the workplace.

Exposure to airborne pollutants and chemicals: Workers in various industries may be exposed to hazardous airborne pollutants and chemicals, such as gases, fumes, and dust. These substances can irritate and damage the respiratory system, leading to the development of COPD over time. Examples include exposure to silica dust in mining or construction, welding fumes, chemical solvents, and diesel exhaust [12,13].

Occupational dust exposure: Dust exposure is a common occupational risk factor for COPD. Mining, construction, and manufacturing workers may be exposed to high levels of dust particles, which can penetrate deep into the lungs and cause chronic inflammation and scarring of the airways. Dust from coal, silica, asbestos, and other materials is particularly problematic and linked to COPD development [14,15].

Smoking and secondhand smoke in the workplace: Smoking remains a significant risk factor for COPD, which is further compounded when individuals smoke. In addition to active smoking, exposure to secondhand smoke in the workplace can also contribute to COPD development among non-smoking workers. This is particularly relevant in industries where smoking is prevalent or workers are exposed to secondhand smoke from customers or colleagues [16-18].

Overview of Industries With High Occupational Risk for COPD in the Indian Subcontinent

Mining and quarrying: Collaborative efforts should address the occupational risks of mining and quarrying. This includes implementing effective dust control measures, such as using water sprays, ventilation systems, and personal protective equipment (PPE), like respirators, to minimize the inhalation of harmful particles. Additionally, promoting regular health surveillance and providing access to proper medical care for workers in these industries can aid in the early detection and management of COPD [5,19].

Construction: Collaboration among researchers, policymakers, and construction industry stakeholders should prioritize implementing preventive measures in the construction sector. This includes enforcing proper respiratory protection, implementing dust control measures like wet cutting and local exhaust ventilation, and providing training on safe work practices and hazard communication. Collaboration can also support developing and adopting alternative construction materials and processes that reduce dust and chemical exposures [20-22].

Textile and garment manufacturing: Collaborative efforts should aim to improve respiratory health in the textile and garment manufacturing industry. This can be achieved by implementing effective engineering controls, such as local exhaust ventilation systems, and using appropriate PPE to reduce exposure to cotton dust, synthetic fibers, and chemicals. Promoting regular cleaning and maintenance of work areas and providing proper training on the safe handling and storage of hazardous substances can also mitigate the risk of COPD [20,23].

Agriculture and farming: Collaboration should prioritize developing and implementing preventive measures in the agricultural sector. This includes promoting good agricultural practices, such as using appropriate respiratory protection, implementing dust control measures, and providing training on the safe handling and application of pesticides. In addition, efforts should focus on raising awareness about respiratory hazards in farming and encouraging regular health checkups for agricultural workers to detect early signs of respiratory diseases, including COPD [24].

The Indian subcontinent faces unique occupational risk factors that contribute to COPD development. Factors such as poor ventilation, inadequate use of PPE, and limited occupational health and safety regulations can exacerbate the impact of occupational hazards [25,26]. In addition to the general occupational risks, region-specific factors may further contribute to COPD prevalence. For example, the prevalence of bidi smoking (a type of tobacco product) is high in India, and workers in the bidi rolling and manufacturing industries are exposed to tobacco dust and smoke, which can increase the risk of COPD. Agricultural workers in the region may also face challenges related to pesticide exposure and using biomass fuels for cooking and heating, further increasing their susceptibility to COPD [25,26].

Understanding these specific occupational risk factors and their contribution to COPD in the Indian subcontinent is crucial for implementing preventive measures and targeted interventions. By addressing these risks through improved workplace safety regulations, better training and awareness programs, and the promotion of healthy work environments, it is possible to reduce the burden of COPD in these industries and protect the respiratory health of workers [25,26].

Occupational Health and Safety Regulations

The Indian subcontinent has established various regulations and policies to address occupational health and safety, including measures to reduce occupational risks associated with COPD. These regulations and policies vary across countries and may include the following.

Occupational Safety and Health (OSH) Act: Countries like India, Pakistan, and Bangladesh have specific OSH Act that outlines legal requirements for workplace safety and health. These acts provide a framework for employers to ensure a safe working environment, including provisions for risk assessments, hazard identification, and control measures [27,28].

National policies and programs: Governments in the region have also implemented national policies and programs focused on occupational health and safety. These policies aim to raise awareness, promote safe working practices, and improve workplace conditions to prevent occupational diseases, including COPD [29,30].

Standards and guidelines: Various standards and guidelines have been developed to regulate occupational exposures. These may include permissible exposure limits for hazardous substances, guidelines for ventilation, and requirements for PPE use [31-33].

Assessment of the Effectiveness of Current Regulations in Addressing Occupational Risks for COPD

The effectiveness of current regulations in addressing occupational risks for COPD in the Indian subcontinent varies. While existing regulations and policies are in place, their implementation and enforcement may face challenges. Some of the key factors influencing the effectiveness of current regulations are as follows.

Enforcement and compliance: Collaborative efforts should prioritize strengthening the enforcement of occupational health and safety regulations. This includes allocating adequate resources, training enforcement personnel, and conducting regular inspections and audits to ensure compliance. Collaborators can work together to identify and address gaps in enforcement, promote the importance of compliance among employers, and establish mechanisms for reporting and addressing non-compliance [34].

Awareness and education: Collaboration is essential for raising awareness and improving education regarding occupational hazards and their health effects. Efforts should focus on developing educational materials, training programs, and awareness campaigns targeting employers, employees, and enforcement agencies. By increasing knowledge and understanding of occupational risks, stakeholders can make informed decisions and take proactive measures to mitigate respiratory hazards and protect workers' health [35,36].

Informal sector: Collaborative efforts should address the unique challenges of the informal sector. This can involve developing targeted strategies to raise awareness, improve access to resources and training, and promote self-regulation among informal sector employers. Collaboration with relevant organizations and associations representing informal sector workers can help facilitate the implementation of occupational health and safety measures [37].

Limited occupational health services: Collaboration among researchers, policymakers, and healthcare providers is crucial for addressing the limited availability and access to occupational health services. This can involve advocating for expanding occupational health services, particularly in rural areas, and integrating occupational health into existing healthcare systems. Collaborators can also work together to develop guidelines and standards for occupational health services and promote the training of healthcare professionals in occupational medicine [38].

Identification of Gaps and Challenges in Implementing and Enforcing Occupational Health and Safety Measures

Despite the existing regulations, several gaps and challenges exist in implementing and enforcing occupational health and safety measures in the Indian subcontinent.

Inadequate regulatory framework: Collaborative efforts should focus on reviewing and updating the regulatory framework to ensure comprehensive coverage of occupational health and safety issues. Stakeholders can work together to identify gaps, address emerging risks, and advocate for developing robust regulations encompassing all industries and evolving work practices [39].

Insufficient resources: Collaboration is essential for advocating increased allocation of resources to occupational health and safety. By pooling resources and advocating for adequate funding, stakeholders can support the implementation and enforcement of regulations, enhance staffing levels, improve infrastructure, and provide necessary equipment and tools to ensure a safe working environment [40].

Informal economy: Collaboration should include engaging and raising awareness among informal sector workers, employers, and organizations. Partnerships with informal sector associations, trade unions, and community-based organizations can facilitate the integration of occupational health and safety measures into informal workplaces. Collaborators can work together to develop tailored approaches, such as simplified guidelines, training materials, and support mechanisms, to address the unique challenges of the informal economy [41].

Limited collaboration: Strengthening stakeholder collaboration is crucial for effectively implementing and enforcing occupational health and safety measures. Collaboration can be fostered through partnerships, interagency coordination, and multi-stakeholder platforms that promote dialogue, information sharing, and joint decision-making. By engaging all relevant parties, including government agencies, employers, workers' organizations, and civil society, collaborative efforts can ensure the alignment of interests and the effective implementation of occupational health and safety measures [42].

Inadequate training and capacity building: Collaboration should prioritize developing and implementing comprehensive training and capacity-building programs. This includes training for employers on risk assessment and management, workers on recognizing and mitigating hazards, and enforcement agencies on effective monitoring and enforcement practices. Collaborators can share resources, expertise, and best practices to develop standardized training materials, conduct workshops, and provide ongoing support to enhance the knowledge and skills of all stakeholders involved [43]. It is important to note that gaps and challenges require concerted efforts from governments, employers, workers, and relevant stakeholders. Strengthening occupational health and safety regulations, improving enforcement mechanisms, enhancing awareness and education, and fostering collaboration can contribute to better protection against occupational risks, including COPD, in the Indian subcontinent.

Impact of COPD on occupational health and the economy

Discussion of the Burden of COPD on Affected Individuals and Their Ability to Work

COPD imposes a substantial burden on affected individuals and their ability to work. The chronic and progressive nature of the disease often leads to symptoms such as breathlessness, coughing, and fatigue, which can significantly impact an individual's quality of life and functional capacity. As COPD progresses, individuals may experience physical activity limitations and face difficulty performing job-related tasks [44].

COPD-related symptoms can interfere with work attendance, productivity, and overall job performance. Flare-ups or exacerbations of COPD symptoms may require frequent medical visits, hospitalizations, and time off work for recovery, leading to increased absenteeism. The persistent symptoms and limitations associated with COPD can result in reduced work productivity, impaired concentration, and decreased efficiency, affecting job performance and career advancement opportunities [45].

Evaluation of the Economic Implications of COPD-Related Productivity Losses

COPD-related productivity losses have significant economic implications at both individual and societal levels. Absenteeism and reduced work productivity due to COPD result in economic losses for individuals, employers, and the economy as a whole. The direct costs associated with medical expenses, hospitalizations, and medication for COPD management contribute to the economic burden [46].

Moreover, COPD-related disability and early retirement can have long-term financial consequences for individuals and their families. Loss of employment or decreased income can lead to financial strain, reduced quality of life, and increased dependence on social support systems [47]. From a societal perspective, the economic burden of COPD extends beyond individual costs. Reduced productivity and absenteeism due to COPD affect the overall productivity of industries and the economy, resulting in decreased national productivity and economic growth.

Importance of Addressing Occupational Risks to Improve Workforce Health and Productivity

Occupational risks associated with COPD are crucial for improving workforce health and productivity. By implementing preventive measures and effective occupational health and safety practices, the incidence of COPD can be reduced, leading to a healthier and more productive workforce [48,49]. Investing in occupational health and safety measures can help mitigate the risk factors contributing to COPD, such as exposure to airborne pollutants, dust, and smoking in the workplace. This includes implementing proper ventilation systems, providing PPE, promoting smoking cessation programs, and raising awareness about the importance of respiratory health [48,49].

By reducing the incidence and severity of COPD in the workplace, employers can create a healthier work environment, enhance employee well-being, and improve productivity. Healthy and productive employees are more likely to remain engaged, contribute effectively to their jobs, and have reduced absenteeism and healthcare costs [48,49]. Furthermore, addressing occupational risks for COPD aligns with broader public health goals and sustainable development agendas. It supports the achievement of targets related to reducing non-communicable diseases, promoting healthy work environments, and enhancing the overall well-being of populations.

Prevention and Mitigation Strategies

Preventing occupational risks associated with COPD requires a comprehensive approach focusing on primary and secondary prevention. The following preventive measures can help reduce the incidence and severity of COPD in the workplace.

Risk assessment and management: Collaborative efforts should focus on conducting comprehensive workplace risk assessments to identify potential respiratory hazards. This involves assessing exposure levels, evaluating the frequency and duration of exposure, and determining the associated health risks. Collaborators can then develop and implement appropriate control measures, such as engineering controls, administrative controls, and PPE, to minimize or eliminate exposure to respiratory irritants and hazardous substances [50,51].

Workplace policies and regulations: Collaboration among researchers, policymakers, and industry stakeholders should aim to develop and enforce robust workplace policies and regulations that prioritize occupational health and safety. These policies may include restrictions on smoking in the workplace, proper handling and storage of chemicals, adequate ventilation requirements, and the use of protective equipment. By establishing and enforcing such policies, employers can create a safe work environment that reduces the risk of COPD and other respiratory diseases [52,53].

Smoking cessation programs: Collaboration efforts should include implementing smoking cessation programs in workplaces to support workers in quitting smoking. These programs can provide resources, counseling, and support to employees who smoke, helping them overcome nicotine addiction and reduce the risk of developing COPD. The promotion of tobacco-free workplaces can create a healthier environment for all workers, reducing the impact of both active smoking and secondhand smoke on respiratory health [54,55].

Health surveillance: Collaboration among researchers, policymakers, and healthcare professionals should emphasize the importance of regular health surveillance programs for workers exposed to occupational hazards. These programs involve periodic health assessments and monitoring to detect early signs of respiratory diseases, including COPD. By implementing health surveillance, potential cases of COPD can be identified early, allowing for timely intervention, treatment, and management of the disease [56,57].

Importance of Early Detection and Diagnosis of COPD in the Workplace

Early detection and diagnosis of COPD in the workplace are vital in preventing disease progression and minimizing its impact on affected individuals and their ability to work. Regular health screenings and medical assessments can help identify respiratory symptoms and impairments early on, allowing for timely intervention and management [58].

Early detection of COPD enables appropriate treatment and the implementation of preventive measures to minimize further lung damage. It allows targeted interventions, such as smoking cessation programs, to reduce the risk factors contributing to COPD development [58]. Moreover, early diagnosis facilitates the implementation of workplace accommodations and adjustments to support individuals with COPD in their job tasks, ensuring a safe and supportive work environment.

Implementation of Engineering Controls, PPE, and Ventilation Systems

Engineering controls, PPE, and ventilation systems are essential strategies for reducing occupational risks for COPD.

Engineering controls: Collaboration efforts should prioritize the implementation of engineering controls to minimize occupational risks for COPD. This includes installing local exhaust ventilation systems to capture and remove airborne pollutants at the source, isolating emission sources to prevent their dispersion into the work environment, and utilizing dust suppression techniques to reduce the generation and dispersal of harmful particles. Implementing these engineering controls can significantly reduce the concentration of respiratory hazards, thereby protecting workers' respiratory health [59].

PPE: Collaboration among researchers, policymakers, and industry stakeholders should emphasize using and providing appropriate respiratory protective equipment (RPE) as part of a comprehensive risk management strategy. This includes providing workers with the necessary RPE, such as respirators, masks, or respirable dust filters, specifically designed to protect against the identified occupational hazards. Collaboration efforts should focus on ensuring that workers are trained on the correct use, maintenance, and limitations of PPE and that employers are responsible for regularly evaluating the effectiveness and adequacy of the provided equipment [60].

Ventilation systems: Collaboration should emphasize the importance of adequate ventilation systems in the workplace. Proper ventilation ensures the supply of fresh air, maintains optimal airflow, and effectively removes contaminants from the work environment. By implementing and maintaining appropriate ventilation systems, employers can dilute and remove hazardous airborne substances, reducing the risk of respiratory irritation and disease. Collaboration efforts should aim to establish guidelines and standards for ventilation system design and operation and educate employers on the importance of regular maintenance and monitoring to ensure their effectiveness [61].

Role of Education and Training Programs for Workers and Employers

Education and training programs are essential components in preventing COPD in the workplace. These programs should target both workers and employers, aiming to enhance awareness, knowledge, and skills related to occupational risks and respiratory health.

Worker education: Collaborative efforts should focus on providing comprehensive education and training to workers regarding occupational hazards and their potential impact on respiratory health. This includes educating workers on the proper use of PPE, safe work practices, and the importance of early reporting of respiratory symptoms. By empowering workers with knowledge and skills, they become active participants in maintaining a healthy work environment and reducing their risk of developing COPD [62].

Employer training: Collaboration should also involve conducting training programs for employers to enhance their understanding of occupational risks and their management. This includes training employers on recognizing and assessing potential hazards, implementing control measures to minimize exposure, conducting regular risk assessments, and establishing protocols for respiratory health surveillance. Equipping employers with the necessary knowledge and tools can create a safer work environment and protect their workers' respiratory health [63].

Promotion of a culture of health and safety: Collaboration among researchers, policymakers, and industry stakeholders should aim to foster a culture of health and safety in workplaces. This involves promoting awareness and understanding of occupational risks and their impact on respiratory health, encouraging open communication channels between workers and management, and involving workers in decision-making processes related to their respiratory health. By creating a supportive and participatory environment, employers can instill a sense of responsibility and ownership among workers, leading to improved adherence to safety protocols and better overall respiratory health outcomes [63]. By investing in education and training programs, employers can promote a better understanding of COPD risks, facilitate the adoption of preventive measures, and create a safer work environment that protects the respiratory health of workers.

Research gaps and future directions

Despite the existing research on occupational risks and COPD in the Indian subcontinent, several gaps in knowledge and understanding still remain. Some of the key research gaps are as follows.

Limited data on occupational COPD: There is a scarcity of studies specifically investigating the prevalence, incidence, and burden of COPD attributable to occupational exposures in the region. More robust epidemiological studies are needed to assess the specific contribution of occupational risks to COPD development and progression [64].

Lack of longitudinal studies: Long-term follow-up studies are essential to examine the cumulative effects of occupational exposures on COPD and understand the latency period between exposure and disease manifestation. Longitudinal studies can also shed light on the effectiveness of preventive measures and the impact of changes in occupational practices on COPD incidence and progression [65].

Occupational risk factors in emerging industries: With the rapid growth of industries and changing work patterns in the Indian subcontinent, there is a need to investigate the occupational risk factors associated with new and emerging industries, such as e-commerce, information technology, and service sectors. Acknowledgment and mitigation of the potential respiratory hazards in these industries are crucial to implementing appropriate preventive measures [66].

Occupational COPD in informal sectors: The informal sector constitutes a significant portion of the workforce in the region, yet research on occupational risks and COPD in this sector is limited. Studies focusing on occupational hazards and COPD in informal sectors, such as small-scale agriculture, construction, and domestic work, are needed to address the unique challenges and develop targeted interventions [67].

Suggestions for Future Research and Studies to Address These Gaps

Prospective cohort studies: Conducting large-scale prospective cohort studies that involve a diverse range of industries and occupations can provide valuable insights into the occupational risk factors for COPD and their long-term effects. These studies should assess exposure levels, use standardized diagnostic criteria for COPD, and collect detailed occupational and health information.

Focus on specific occupations and exposures: Investigating high-risk occupations and exposures prevalent in the Indian subcontinent, such as mining, construction, and textile manufacturing, can help identify the magnitude of risks and develop targeted prevention strategies.

Assessing interventions and control measures: Evaluating the effectiveness of existing occupational health and safety interventions, including engineering controls, PPE, and workplace policies, in reducing COPD risk and improving respiratory health is crucial. Comparative studies can help identify best practices and guide the implementation of effective preventive measures.

Socioeconomic and cultural factors: Exploring the influence of socioeconomic and cultural factors on occupational risks and COPD in the Indian subcontinent is important for a comprehensive understanding. Factors such as education, income disparities, gender inequalities, and access to healthcare may influence exposure levels, disease progression, and health-seeking behaviors.

Importance of Collaborative Efforts Between Researchers, Policymakers, and Industry Stakeholders

Collaborative efforts between researchers, policymakers, and industry stakeholders are vital to advancing knowledge, promoting effective interventions, and implementing policies to address occupational risks and COPD. Collaboration can foster the following.

Data sharing and standardization: Collaboration is crucial in data sharing and standardization. By collaborating, researchers can pool their data, establish common methodologies, and develop standardized approaches for assessing occupational risks and COPD. This facilitates the comparability of studies and ensures that research findings can be combined and analyzed effectively, leading to more robust evidence and better-informed decision-making [68].

Policy development and implementation: Collaboration between researchers and policymakers is vital for translating research findings into meaningful policies and regulations. Through collaboration, policymakers can access the latest research evidence on occupational risks and COPD, which can inform the development of effective policies and regulations. Furthermore, collaboration ensures these policies are implemented and enforced properly, improving workplace occupational health and safety practices [69].

Industry engagement and adoption of best practices: Collaborating and initiating with industry stakeholders, including employers, trade unions, and professional organizations, are essential for promoting best practices to mitigate occupational risks. By engaging with these stakeholders, researchers can gain insights into the practical challenges faced by industries and work together to develop and implement effective preventive measures. This collaborative approach fosters a culture of health and safety in the workplace and encourages industries to prioritize the well-being of their workers [70].

Translation of research into practice: Collaboration helps bridge the gap between research findings and their application in real-world settings. By engaging in knowledge exchange platforms, workshops, and capacity-building initiatives, researchers can interact directly with stakeholders and facilitate the translation of research outcomes into practical interventions and policies. This collaborative effort ensures that research findings are effectively communicated, understood, and implemented in occupational health and safety practices, ultimately improving the respiratory health of workers in the Indian subcontinent [71].

## Conclusions

In conclusion, addressing occupational risks and reducing the burden of COPD in the Indian subcontinent necessitate collective action and collaboration. The importance of strengthening occupational health and safety regulations is paramount, with policymakers focusing on their implementation and enforcement across industries. This includes updating regulations to address emerging risks and ensuring compliance through regular inspections and monitoring. Enhanced research efforts are vital for comprehensively understanding occupational risks and COPD in the region. Prospective studies, specific occupations and exposures, interventions, and socioeconomic factors should be the focus, providing robust evidence to guide preventive strategies and inform policies. Education and awareness are crucial for raising consciousness among workers and employers about occupational risks and fostering a culture of health and safety. The development of education and training programs can improve knowledge, skills, and practices related to respiratory health and occupational risk prevention. Collaboration among researchers, policymakers, industry stakeholders, and healthcare professionals is key. By working together, evidence-based policy-making, the adoption of best practices, and the implementation of preventive measures can be achieved in workplaces. Therefore, prioritizing occupational health and safety, implementing effective preventive measures, and promoting a culture of respiratory health in the Indian subcontinent are essential. These actions contribute to creating healthier work environments, protecting workers' well-being, and mitigating the burden of COPD associated with occupational risks.
